# The Teensleep study: the effectiveness of a school-based sleep education programme at improving early adolescent sleep

**DOI:** 10.1016/j.sleepx.2019.100011

**Published:** 2019-12-07

**Authors:** Gaby Illingworth, Rachel Sharman, Christopher-James Harvey, Russell G. Foster, Colin A. Espie

**Affiliations:** Sleep and Circadian Neuroscience Institute, Nuffield Department of Clinical Neurosciences, University of Oxford, UK

**Keywords:** Sleep education, School-based intervention, Adolescent sleep, Sleep knowledge, Behaviour change, Actigraphy

## Abstract

**Objective:**

To evaluate the impact of a school-based sleep education programme on adolescent sleep and sleep knowledge.

**Methods:**

This is the first outcome report on ‘Teensleep’: a novel, teacher-led programme, comprising ten lessons that can be delivered flexibly. Students in Year 10 (*n* = 1504; mean age = 14.14 ± 0.35 years) from ten UK state (non-fee-paying) secondary schools received the lessons and parents received a leaflet. Effectiveness was assessed using measures across two time points (pre- and post-intervention). Students completed questionnaires on sleep knowledge, sleep quality, sleep behaviour, sleep hygiene, daytime sleepiness and health-related quality of life. A sub-sample provided objective (actigraphy, *n* = 84) and subjective (sleep diary, *n* = 74) sleep measures.

**Results:**

Large improvements in sleep knowledge (*d* = 0.78), and smaller improvements in sleep quality (*d* = 0.15) and sleep hygiene (*d* = 0.11) were observed, but not in daytime sleepiness or health-related quality of life. Small and limited changes in subjective and objective sleep patterns were found. Baseline sleep quality was differentially associated with key outcomes, with those initially self-reporting poor sleep demonstrating an improvement in sleep quality, sleep hygiene and sleepiness.

**Conclusion:**

Teensleep was effective at improving sleep knowledge but sleep changes were small. Such interventions have traditionally focused on gains for all students, but this study suggests that poor sleepers may be the most likely to experience immediate direct sleep benefits. Follow-up studies are required to investigate whether or not sleep education provides long-term benefits as a step towards preventative sleep medicine.

## Highlights

• ‘Teensleep’ is a teacher-led, school-based sleep education programme for adolescents.
• Large-scale study: students received lessons in ten UK schools.
• Programme effectiveness was evaluated using questionnaires and actigraphy.
• Sleep knowledge, self-reported sleep quality and sleep hygiene improved after lessons.
• Study suggests poor sleepers may be most likely to experience immediate benefits.

## 1 Introduction

Adolescents face a number of challenges in getting a good night's sleep arising from both endogenous and exogenous factors. It is well established that the biological regulation of sleep undergoes a developmental change in adolescence, demonstrated by a shift in sleep-wake patterns. The circadian timing system delays, with an accompanying preference for later bed and wake times [[1], [2], [3]]. In addition, a slower accumulation of homeostatic sleep pressure contributes to the likelihood that adolescents will still feel alert and awake later in the evening [4,5].

As well as these bioregulatory factors, a multitude of environmental, psychosocial and lifestyle factors influence the duration, quality and timing of adolescent sleep. Consequently, adolescents experience sleep that is not only of insufficient duration but is also inappropriately timed [6]. School start times may contribute to short sleep during the school week, leading to a build-up of ‘sleep debt’ with ‘catch-up sleep’ at the weekends. A review of adolescent sleep worldwide found that weekend sleep practices of later bedtimes and morning lie-ins are highly prevalent [7]. This tendency to keep inconsistent sleep schedules can further exacerbate the delayed sleep-wake cycle. Psychosocial influences such as a desire for increased autonomy with a reduction in parental control over bedtimes [8,9], social stresses [10] and homework and extracurricular activities [11] can play a role in delayed or problematic sleep. Furthermore, lifestyle factors such as caffeine consumption [12] and using electronic media before bedtime [[13], [14], [15]] can interfere with and shorten sleep by encouraging later bedtimes and increasing arousal at night [16]. Conversely, good sleep hygiene practices appear to be a protective factor for adolescent sleep [17].

A concerning global pattern has emerged of delayed sleep-wake behaviour, daytime sleepiness, and short sleep [7,18,19] with a downward trend over time in self-reported sleep duration in adolescence [20,21]. Although there is debate about how much sleep adolescents need [22], the National Sleep Foundation guidelines recommend that 14- to 17-year-old adolescents should be sleeping between eight to 10 h a night [23]. However, a meta-analysis showed that teenagers were getting less than 8 h’ sleep on a school night in 53% of samples whilst a review noted that daytime sleepiness was a prevalent problem [7]. It is important to address poor sleep in adolescents because of the fundamental role that sleep plays in learning, memory and emotion regulation and evidence that insufficient and poor quality sleep, as well as excessive sleepiness, are associated with impairments in adolescent functioning [[24], [25], [26], [27]]. Moreover, an improvement in adolescent sleep quality or patterns may protect against poor health-related quality of life (HRQoL) [28].

In recognition of these matters, there is growing consensus about, and momentum towards, prioritising sleep in school-based education; just as, for example, physical activity and diet have become standard parts of the health and wellbeing curriculum. A review of 15 published studies found a variety of sleep-related goals for these programmes including: increasing sleep knowledge and/or improving sleep behaviour; changing sleep behaviour, decreasing daytime sleepiness and improving functional outcomes; and increasing awareness of the importance of sleep as well as prioritising sleep hygiene [29]. However, reviews have noted that although programmes have generally been effective at enhancing sleep knowledge, this has not usually led to a change in sleep behaviour [[29], [30], [31]]. To increase the likelihood of behavioural change, reviews of the literature have recommended some putative beneficial components, such as considering the influence of others by involving parents and peers in the process. For example, programmes could seek to increase parental awareness about the importance of sleep and the need for change, as well as involving them in creating healthy sleep environments and providing them with educational materials [[30], [31], [32]]. Above all, the inclusion of motivational elements within a programme has been highlighted as particularly important and key to successful facilitation of behaviour change [30,31].

Sleep behaviour changes have included: increases in sleep duration and time in bed; advances in bedtime; improvements in sleep hygiene; and decreases in sleep onset latency and school day/weekend sleep schedule irregularity [[33], [34], [35], [36]]. Studies have primarily assessed sleep using solely subjective measures but the addition of objective methods has been recommended [37]. However, only a few studies have utilised actigraphy with adolescents, and sometimes with small samples [38,39]. In a larger, randomised controlled trial, Rigney and colleagues [34] investigated whether a school-based programme would improve the sleep of 175 participants monitored by actigraphy, and found that the intervention group delayed their wake time by 10 min immediately after the intervention but not at a 12-week follow-up assessment. To our knowledge, no peer-reviewed studies to date have assessed the effectiveness of a sleep education programme within schools in the U.K.

The current study examined the efficacy and feasibility of the ‘Teensleep’ sleep education programme developed as a school-based intervention for mid secondary school students (14- to 15-year-olds) in the U.K. This large pilot study was designed to assess the Teensleep intervention as part of the decision-making process in whether the intervention would subsequently be assessed via a randomised controlled trial. The primary aim of the study reported here was to evaluate the effectiveness of a sleep education programme at improving adolescent sleep, specifically whether it would be linked to increased sleep duration, improved sleep quality, and reduced sleep pattern variability, and to do so using both objective and subjective measures of sleep. Further aims were to determine whether the intervention improved sleep knowledge and sleep hygiene, and to evaluate whether sleep education lessons would be linked to an improvement in functional outcomes, specifically reduced daytime sleepiness and improved HRQoL.

## 2 Methods

### 2.1 Study design and participants

The study was uncontrolled, comprising one intervention (Teensleep), comparing pre- and post-intervention values of primary and secondary measures. Students in Year 10 from ten state secondary schools in the U.K. participated in the study between September and December 2016 (*n* = 1504; mean age = 14.14 ± 0.35 years; 54 per cent female). Schools were located in areas representing varied socio-economic status backgrounds. The proportion of students who were currently receiving free school meals across the school (FSM; eligibility for FSM is a proxy for socio-economic status in England and Wales) ranged from 0.8 to 28.6 per cent. Two schools had a selective admissions policy and all but one were co-educational, the exception being an all girls' school. All students in the year group received the lessons and were asked to provide sleep knowledge quiz and survey data unless parents/caregivers had returned opt-out forms. Opt-in parental/caregiver and student consent was required for the collection of actigraphy and sleep diary data in a self-selecting sub-sample of 15–20 students per school. The availability of actiwatches informed the size of the sub-sample (*n* = 160). A student was excluded from this sub-sample if they had a current sleep disorder, psychological or physiological disorder that might interfere with sleep, took regular medication that might impact sleep, suffered a concussion where they were unconscious for more than 5 min, or had been to a country which was three or more time zones from the UK within the last six months. A separate evaluation of the study, including acceptability and feasibility, was carried out in accordance with a requirement specified by the Education Endowment Foundation (EEF), a co-funder. For this information, please see the evaluation report by Durham University (educationendowmentfoundation.org.uk). The study was approved by the University of Oxford Central University Research Ethics Committee and Durham University's School of Education Ethics Committee.

### 2.2 Procedure

#### 2.2.1 The sleep education programme

The Teensleep programme aims to increase students’ knowledge and understanding of sleep, and to facilitate sleep-related behavioural change, by improving sleep hygiene, providing techniques to deal with stress/the racing mind, and increasing motivation to establish a consistent sleep pattern. Interventional elements were adapted for classroom use from cognitive behaviour therapy (CBT), known to improve sleep in people with insomnia [c.f., [40,41]]. The programme was developed collaboratively between our sleep group in Oxford and teaching professionals. The initial programme was piloted in six schools in early 2016. Feedback incorporating separate focus groups of teachers and students led to amendments to lessons and study materials including improving accessibility/engagement and relevance to the teenage years.

Our purpose was to agree a package that could be delivered efficiently and effectively, in accordance with school requirements, yet also flexibly so that schools could be pragmatic about how they incorporated Teensleep into their timetable. The final programme comprised 5 h' material, across ten lessons: three lessons dedicated to the science of sleep; four to sleep hygiene; and three to stress management. Lesson topics are summarised in Table 1. The lessons were designed to be discursive, interactive, and self-reflective, with a focus on the students and their own sleep. Teacher workbooks guided the structure and delivery of each lesson with accompanying PowerPoint slides. Every student received a workbook including information and activities for each lesson. Students were to write in these to create a personalised sleep book. For example, an activity entitled ‘Your take home message’ was designed to encourage students to individualise information from each lesson which could act as knowledge reflection for the next lesson. The lessons promote positive attitudes towards sleep, encourage self-efficacy and address barriers to sleep (eg, use of electronic devices) whilst considering social influence (eg, peer engagement in the sleep education programme).

**Table 1:** Teensleep lessons.

Lesson	Topic	Lesson content
1	What is sleep?	Sleep quiz; Describe what is sleep; Recognise the difference between good and poor sleep
2	Why is sleep important?	Summarise the important role sleep plays in their lives; Identify the effects of poor sleep
3	The body clock and sleep drive	Talk about what controls when we sleep and how it changes in teenagers; Say whether they are an “Early bird”, a “Night owl”, or someone in between; Discuss how the timing of their alertness has changed from aged ten to now
4	Sleep scheduling	Work out their own sleep schedule; Use sleep scheduling to improve their sleep; List things that can change how they sleep
5	Lifestyle and sleep	Recognise lifestyle factors that can influence sleep
6	Light and sleep	Understand the impact of light and sleep; Tell someone how electronic devices can disrupt sleep
7	Creating a sleep haven	Assess the sleep-friendliness of their bedroom; Create a link between their bed and sleep
8	Thoughts and emotions at bedtime	Identify thoughts and emotions that might interfere with sleep; Practise breathing exercises to relax
9	Managing the racing mind	Recognise and work on restructuring unhelpful thoughts; Practise progressive muscle relaxation
10	The sleep friendly routine	Practise thought-blocking to deal with repetitive thoughts; Practise a visualisation tool to reduce mental alertness at night; Plan their bedtime routine to help sleep; Sleep quiz

To encourage behaviour change, regular prompts and opportunities for students to devise action plans based on what they had learnt were core components. Lessons included ‘Give it a go’ activities (eg, moving bedtimes and wake times to match their ideal sleep schedule over a weekend; turning electronic devices off or on to silent mode overnight; avoiding using electronic devices in the hour before sleep; practising progressive muscle relaxation for a few nights). Students were asked to reflect on what might stop them doing the activity and to think about how they could overcome these potential obstacles to make success more obtainable. Students were prompted to share what they did and whether the behaviour helped their sleep in order to consider peer influence and encourage adherence to their chosen action. Focus groups were conducted by the evaluation team from Durham University to gather qualitative feedback from students regarding the acceptability of the programme. This is presented elsewhere, in the evaluation report mentioned previously (educationendowmentfoundation.org.uk).

#### 2.2.2 Teacher training

Teacher training was conducted at each school by two postdoctoral researchers who had developed the lessons. The 3-h training session was supported by a PowerPoint presentation and each teacher received a study pack including workbooks and study materials. Teachers who received the training, supported by relevant training resources, cascaded it to staff who were not able to attend. An assessment of teacher training was carried out by the evaluation team comprising observations of training sessions, a teacher survey and interviews, and is detailed in the evaluation report (educationendowmentfoundation.org.uk). Although sleep knowledge was not formally tested after the training, it was considered that teachers had received sufficient information to deliver the lessons.

#### 2.2.3 Parent leaflet

A leaflet summarising the lessons' key messages was provided for parents in order to improve sleep hygiene knowledge and increase the priority given to sleep in the family, and so parents might encourage their child's engagement with the programme and support any behaviour change that students might try to implement. Schools were to distribute leaflets after the first lesson so as not to influence students' baseline knowledge of sleep.

#### 2.2.4 Evaluation

Students were asked to fill the surveys out twice: in the week prior to the lessons beginning (pre-intervention assessment) and again during the second week following the last lesson (post-intervention assessment). A sleep knowledge quiz was taken twice, once at the beginning of lesson 1 and again at the end of lesson 10. This was a surprise quiz so students were not asked to revise the information they had been taught. The sub-sample of students involved in more comprehensive sleep monitoring was asked to wear the actiwatch and complete the sleep diary for 14 continuous days both before the lessons began and immediately after the lessons had finished. The researchers conducted screening interviews with those students wishing to take part in sleep monitoring and explained how to use the actiwatches and complete the sleep diaries. In the event that there were more eligible students than available actiwatches, students were randomly selected to take part. Within each school, students who wore an actiwatch for 14 nights or completed the survey at either time point were entered into a prize draw to win Amazon gift vouchers. Students who provided a complete set of actigraphy data (total of 28 nights) were entered into a prize draw across all schools to win a 32 GB iPad Pro.

### 2.3 Measures

#### 2.3.1 Sleep knowledge

Sleep knowledge was measured using a 20-question multiple choice quiz. This format was used to reduce the possibility of ceiling effects that may have explained low sensitivity to change with some sleep knowledge tools incorporating True/False responses [37]. A point was awarded for each correct response and a total score obtained by summing all correct responses (0–20). The quiz was developed by the authors for the purposes of this study, with all questions based on information provided in the lessons. Eight questions related to sleep science (eg, ‘At puberty, a teenager's biological urge to sleep delays by … ’ and twelve questions covered sleep hygiene (eg, ‘Which of the following would be a good bedtime routine?‘) and stress management (eg, ‘Tensing and relaxing muscles in the body to help us relax is known as … ‘).

#### 2.3.2 Survey data

The survey was completed using pen and paper due to concerns over limited access to computers within schools.

##### 2.3.2.1 Sleep quality

The Sleep Condition Indicator (SCI) [42], a brief measure of insomnia symptoms, was used to assess sleep quality over the previous two weeks. The original questionnaire asks about sleep over the last month but the timescale was adapted due to post-intervention measurement taking place within two weeks of the last lesson. It comprises 8 items with each item rated on a five point scale of zero to four. A total score (0–32) is obtained by summing all responses. A higher score indicates better sleep quality whilst a lower score indicates greater symptom severity. Although cutoffs are based on adults rather than adolescents, a score of ≤16 was considered to indicate ‘probable insomnia disorder’. The SCI had good internal consistency in this sample of adolescents (baseline: *n* = 1033; Cronbach's α = 0.84).

##### 2.3.2.2 Sleep behaviour

The Munich Chronotype Questionnaire (MCTQ) [43] was used to provide sleep timing, duration and efficiency. Weekday and weekend timings were reported separately. Sleep duration was calculated from the difference between sleep onset (calculated using ‘get ready to fall asleep at’, hereafter called ‘lights out’, and ‘minutes to fall asleep’ variables) and wake time, hereafter called ‘lights on’. Sleep efficiency was calculated as sleep duration expressed as a percentage of time in bed after lights out and before lights on (sleep intention window). Lights out was used rather than bedtime to allow for the possibility that teenagers may spend time in bed working/using social media rather than having sleep intention, which might then lead to an artificially reduced sleep efficiency.

##### 2.3.2.3 Sleep hygiene

The Adolescent Sleep Hygiene Scale – Revised (ASHS-r) [44] was used to measure how often sleep hygiene behaviours or events occurred during the previous two weeks (timescale adapted as per SCI – see above). This comprises 24 items, with each item rated on a 6-point scale (from 1 [never, 0%] to 6 [always, 100%]). Scores are reversed so higher scores indicate better sleep hygiene. The ASHS-r has six subscales: physiological (five items), behavioural arousal (three items), cognitive/emotional (six items), sleep environment (five items), sleep stability (three items), and daytime sleep (two items). Each subscale score is the mean of all its items. The total sleep hygiene score is the mean of all subscale scores.

##### 2.3.2.4 Daytime sleepiness

The Cleveland Adolescent Sleepiness Questionnaire (CASQ) [45] was used to measure daytime sleepiness during a usual week. This comprises 16 items, with each item rated on a 5-point scale (from 1 [never, 0 times per month] to 5 [almost every day, 5 or more times per week]). Items measure sleepiness (11 items) and alertness (five items; reverse scored) which are then summed for a total sleepiness score. Higher scores indicate greater sleepiness.

##### 2.3.2.5 Health-related quality of life

Kidscreen-27 (KS-27) [46] was used to measure HRQoL, conceptualised as a multidimensional construct encompassing physical, emotional, mental, social and behavioural dimensions of wellbeing and functioning. This comprises 27 items, with each item answered on a 5-point scale assessing frequency or intensity. Five dimensions cover physical wellbeing (five items), psychological wellbeing (seven items), autonomy and parent relations (seven items), social support and peers (four items), and school environment (four items). Higher scores indicate better HRQoL. Recommended syntax was used to compute Rasch person parameter estimates from summed scale values that are subsequently transformed into T-values with a mean of 50 and a standard deviation of 10.

#### 2.3.3 Sleep assessment by actigraphy and sleep diary

##### 2.3.3.1 Actigraphy

Actigraphy was used to provide an objective measurement of sleep. Participants wore a MotionWatch 8 device (MW8; CamNtech Ltd, Cambridge, U.K.). These telemetric devices are worn on the non-dominant wrist, using movement to determine rest/activity cycles. Data were recorded at 30-s epochs in accordance with the validated sleep algorithm, and analysed using the inbuilt sleep software. Students were asked to press an event marker button on the device when they were in bed, had switched off the lights, and were attempting to sleep, and again when they woke up in the morning and had no intention of returning to sleep. As actigraphy uses movement to determine sleep onset/offset, this method was considered the most accurate way to differentiate sleep intention from, for example, lying in bed using social media. Each sleep period over the 14 days was manually evaluated. The markers for ‘fell asleep’ and ‘woke up’ (final awakening) were automatically adjusted according to the event markers and the activity data. If the student forgot to press the event marker, the sleep diary time was used instead, and if there was no corresponding diary day, or activity was significantly discrepant from the event marker or diary, then the markers were placed when activity ended or began (in this instance, sleep latency data were consequently not included). The sleep variables were calculated separately for weekday and weekend and averaged over the number of nights provided to yield habitual sleep patterns. These included ‘lights out’ (time the participant put the lights out and tried to sleep) ‘lights on’ (time the participant woke up at the end of the main sleep period and had no intention to sleep longer), sleep onset latency (SOL; time between ‘lights out’ and ‘fell asleep’), total sleep time (TST; total time spent in sleep according to the epoch-by-epoch wake/sleep categorisation), wake after sleep onset (WASO; total time spent in wake according to the epoch-by-epoch wake/sleep categorisation), and sleep efficiency (SE; total sleep time expressed as a percentage of time in bed after lights out and before lights on). Sleep pattern variability was calculated by taking the absolute difference from each weekday or weekend variable (for lights on and lights off) from the relevant average weekday variable and calculating the mean difference.

##### 2.3.3.2 Sleep diary

A sleep diary was used to provide a subjective measurement of sleep as well as to validate the scoring of actigraphy. Each day included questions about sleep timing, quantity and quality. Sleep pattern variability was calculated following the same procedure as above (see Section 2.3.3.1). Sleep hygiene behaviours focused on caffeine intake after 6pm, recorded as percentage of days where students consumed high caffeine items (listed as coffee, energy drink), or low caffeine items (listed as tea, chocolate, fizzy drink) from number of days reported. Electronic media use in the hour before bed was also recorded as a percentage of days used from number of days reported (listed as use of laptop, tablet, PC, mobile phone, e-reader, games console, TV). Sleep quality and cognitive/physical factors that could potentially affect sleep were assessed using four items rated on a 5-point scale (from 0 [not at all] to 4 [very]), asking how rested/refreshed and how awake they felt that morning, and how mentally alert or physically tense they were in bed the previous night. To measure whether participants perceived they had experienced sufficient sleep, they were asked if they would have liked to sleep longer if they could and if so, for how much longer. All measures were averaged over the number of nights provided.

### 2.4 Statistical analyses

Analyses reported focus on changes from pre-intervention (Time 1; T1) to post-intervention (Time 2; T2). Although all students in Year 10 received the sleep education lessons as part of the school timetable (*n* = 1504), participation in study measures was voluntary. A total of 1493 students provided some data for the survey (T1: *n* = 1258; T2: *n* = 1106) and/or the quiz (T1: *n* = 1094; T2: *n* = 970, one school mislaid the second quiz so the return rate was lower). Questionnaires were included in analyses if participants had provided an answer for every item in that questionnaire (for sample sizes, see Table 2), so that subscale and/or total scores could be calculated from all relevant items. The MCTQ was the exception to this as the questionnaire does not comprise subscale/total scores. Completion and retention rates varied by questionnaire. The percentage of students who completed a questionnaire at both time points from all those who received the lessons were as follows: SCI: 46%; MCTQ: ∼45%; CASQ: 48%; ASHS-r: 37%; KS-27: 47%. Consequently, sample sizes included in analyses were different depending on the measure. Of the 160 students selected to take part in the sub-sample sleep monitoring, 16 were excluded from the final sample because they did not provide appropriate consent. Students' actigraphy and/or sleep diary data were excluded from analysis unless a minimum of seven days' actigraphy/fully-completed sleep diary (incorporating five weekdays, ie, Sunday to Thursday night, and two weekend days, ie, Friday and Saturday night) were provided at both time points. A total of 98/89 students (actigraphy/sleep diary) met this requirement but due to time restrictions when data collection ran into the holidays at two schools (and thus weekday actigraphy/sleep diary did not represent a typical school day), a further 13/15 students' data were excluded. One participant's actigraphy was excluded due to a time shift error with the data. Therefore, 84/74 students were included in pre–post actigraphy/sleep diary analysis and of these, 61 students provided both actigraphy/sleep diary data. The overall percentage of students who provided sufficient data for pre–post analysis from the number that consented to take part (*n* = 144) was 58% for actigraphy, 51% for the sleep diary, and 42% for both measures.

**Table 2:** Means and standard deviations for quiz and survey variables (sleep knowledge, sleep quality, sleep behaviour, daytime sleepiness, sleep hygiene and HRQoL) at Time 1 and Time 2.

Variable	Measure	N	T1 M (SD)	T2 M (SD)
Sleep knowledge***	Quiz	779	9.85 (2.83)	12.18 (3.27)
Sleep quality***	SCI	694	23.15 (6.55)	23.87 (5.96)
Weekday TST (hh:mm)**	MCTQ	656	7:25 (1:17)	7:34 (1:13)
Weekend TST (hh:mm)	MCTQ	617	8:58 (1:42)	9:03 (1:41)
Weekday SE (%)**	MCTQ	655	93.39 (7.77)	94.12 (5.85)
Weekend SE (%)	MCTQ	616	95.45 (5.32)	95.46 (5.68)
Weekday SOL (min)	MCTQ	770	29.89 (31.87)	27.78 (30.76)
Weekend SOL (min)	MCTQ	746	25.84 (32.14)	25.83 (32.45)
Daytime sleepiness*	CASQ	727	35.73 (9.71)	36.27 (9.36)
Sleep hygiene total score**	ASHS-r	562	4.19 (0.68)	4.26 (0.65)
HRQoL: Physical wellbeing	KS-27	710	44.03 (8.63)	43.61 (8.33)
HRQoL: Psychological wellbeing	KS-27	710	44.44 (9.38)	44.25 (9.39)
HRQoL: Autonomy & parent relations	KS-27	710	47.70 (10.60)	47.54 (10.52)
HRQoL: Social support & peers***	KS-27	710	48.76 (9.96)	46.63 (10.52)
HRQoL: School environment	KS-27	710	44.66 (8.52)	44.59 (9.08)

*Note:* Discrepancies in sample size are due to missing responses; SCI = Sleep Condition Indicator; MCTQ = Munich Chronotype Questionnaire; CASQ = Cleveland Adolescent Sleepiness Questionnaire; ASHS-r = Adolescent Sleep Hygiene Scale – revised; HRQoL = Health-related quality of life; KS-27 = Kidscreen-27. *p < 0.05, **p < 0.01, ***p < 0.001.

Additional analyses were conducted involving subgroups to evaluate the potential impacts of the intervention on sleep outcome variables depending on how students rated their sleep quality at baseline. It was conceivable that those who perceived they were sleeping well before the lessons, might be less likely to report changes in survey measures after the lessons. Students were grouped by SCI cut-offs into those with/without probable insomnia disorder (≤16/>16) at T1 and were included in analyses if they completed the SCI at both timepoints (≤16, *n* = 113; >16, *n* = 581).

Quantitative data analyses were conducted using the Statistical Package for the Social Sciences (IBM SPSS Statistics Version 25.0, USA). To examine the impact of the intervention, paired-samples *t*-tests were used to investigate differences between pre–post measures. Effect size was calculated using Cohen's *d* and was interpreted as small (*d* = 0.2), medium (*d* = 0.5) and large (*d* = 0.8) [47]. A *p*-value <0.05 was considered to indicate statistical significance. Due to the exploratory nature of the study, adjustments to the alpha level were not made for multiple comparisons in the sample, in order to reduce the risk of Type II errors.

## 3 Results

### 3.1 Sleep knowledge, survey sleep outcome variables and HRQoL

An overview of means and standard deviations (SDs) for these outcome variables from T1 to T2 can be found in Table 2, with full details available in Supplementary Table 1. Paired-samples *t*-tests revealed a significant improvement in sleep knowledge [*t* (778) = −21.90, *p* < 0.001; *d* = 0.78], and a significant improvement in sleep quality [*t* (693) = −3.94, *p* < 0.001; *d* = 0.15]. However, students reported a small increase in daytime sleepiness [*t* (726) = −2.05, *p* = 0.04; *d* = 0.08].

Students reported significant changes in sleep, specifically that they slept 9 min longer [*t* (655) = −3.17, *p* = 0.002], slightly improved sleep efficiency [*t* (654) = −2.69, *p* = 0.007] and put their lights on 3 min later [*t* (797) = −3.30, *p* = 0.001]. There were no significant differences in weekday SOL or lights out. Regarding sleep at the weekend, the only significant change found was in lights out, which was 8 min earlier after the intervention [*t* (684) = 2.69, *p* = 0.007].

Students significantly improved their sleep hygiene practices for the total score [*t* (561) = −2.61, *p* = 0.009; *d* = 0.11] and for the cognitive/emotional [*t* (561) = −2.44, *p* = 0.02; *d* = 0.10] and sleep stability [*t* (561) = −2.93, *p* = 0.004; *d* = 0.12] subscales. There were no changes in HRQoL apart from a marginal worsening in the social support and peers subscale [*t* (709) = 5.52, *p* < 0.001; *d* = 0.21].

### 3.2 Subgroup evaluation based on SCI cutoff

Before the intervention, the majority of students (84%) reported their sleep quality (SCI) was above the cutoff (higher scores indicate better sleep quality) and not indicative of probable insomnia (*n* = 581, *M* = 25.40, SD = 4.05). These students reported no significant change in sleep quality (*p* = 0.50) with 95% remaining in this category at T2, nor did they report a significant change in sleep hygiene (*n* = 454, *p* = 0.18) measured using the total score of ASHS-r. However, as with the overall sample, these students reported an increase in daytime sleepiness measured using CASQ (T1: *M* = 33.75, SD = 8.38; T2: *M* = 35.04, SD = 8.69) [*t* (566) = −4.77, *p* < 0.001; *d* = 0.20].

Considering students who self-reported poor sleep (16%) at baseline (*n* = 113, *M* = 11.54, SD = 4.23), there was a significant improvement in sleep quality at T2 (*M* = 16.53, SD = 6.68) [*t* (112) = −8.72, *p* < 0.001; *d* = 0.82]. Of these, 52% (*n* = 59) changed to self-report outside the cutoff for probable insomnia. In addition, students reported significantly better sleep hygiene (T1: *M* = 3.72, SD = 0.79; T2: *M* = 3.94, SD = 0.75) [*t* (86) = −2.57, *p* = 0.01; *d* = 0.28], as well as a reduction in daytime sleepiness [T1: *M* = 45.37, SD = 9.65; T2: *M* = 42.53, SD = 9.77] [*t* (117) = 3.60, *p* < 0.001; *d* = 0.33].

### 3.3 Sub-sample actigraphy and sleep diary measures

In line with the sample as a whole, a high proportion of the actigraphy sub-sample rated their sleep quality as not indicative of insomnia at the outset using SCI cut-offs (*n* = 53; 83%), compared with those with poor sleep (*n* = 11; 17%). Paired-samples *t*-tests revealed a significant reduction in subjective weekday WASO [*t* (73) = 2.71, *p* = 0.008; *d* = 0.32], as well as a 5-min delay in objective lights on [*t* (83) = −2.72, *p* = 0.008] and a 6-min delay in subjective lights on [*t* (73) = −3.45, *p* = 0.001] for a weekday. However, TST, SE, SOL, and lights out yielded no significant differences (*t*s < −1.78, *p*s > 0.09) as recorded by actigraphy or sleep diary. The only measure of sleep pattern variability to demonstrate a significant difference was objective weekday lights out variability which increased by 4 min [*t* (83) = −2.46, *p* = 0.016]. For descriptive statistics, see Supplementary Table 2.

Regarding sleep hygiene behaviours, there were no significant differences in caffeine consumption or electronic media use (*t*s < 1.41, *p*s > 0.16). With respect to perceived sleep need, there were no significant changes in the percentage of nights recorded by students as those when they would have liked to sleep longer nor in the average time they would like to have slept longer over all nights recorded (*t*s < 0.94, *p*s > 0.35). Students reported a weekday improvement in how rested/refreshed they felt [*t* (73) = −2.28, *p* = 0.03; *d* = 0.26] as well as a reduction in how mentally awake they were in bed [*t* (73) = 2.39, *p* = 0.02; *d* = 0.28]. However, no other significant changes in sleep quality or cognitive/physical factors that could potentially affect sleep were revealed (*t*s < 1.39, *p*s > 0.16). For descriptive statistics, see Supplementary Table 3.

## 4 Discussion

The current study evaluated the impact of a school-based sleep education programme on objective and subjective sleep, as well as a range of other measures including sleep knowledge, sleepiness, sleep hygiene and HRQoL, in a large sample of adolescents. The greatest change was observed in students’ sleep knowledge, an improvement consistent with most [33,35,48,49], but not all [34] sleep education interventions. Therefore, the study demonstrated that students were able to internalise information about sleep from teacher-led lessons; an encouraging finding if sleep education programmes are to be scalable within schools. Also in line with an earlier study [36], self-reported sleep hygiene improved following the lessons. Although speculative at this stage, the gain in knowledge about sleep hygiene and stress management may have been translated into the small improvements reported. Promisingly, self-reported sleep quality according to SCI ratings improved, albeit with a small effect size.

As has been found with previous studies [[49], [50], [51]], this study further highlights how changing adolescent sleep behaviour through sleep education is challenging. Small improvements in students’ subjective sleep duration and sleep patterns as measured by questionnaire were evident; for example, students reported an average 9-min increase in weekday TST from 7:25 h to 7:34 h. While positive, this is less than the 27-min increase in TST reported by Bonnar and colleagues [33]. Albeit in the right direction, these findings may not represent a change of clinical interest and self-reported sleep duration remained below National Sleep Foundation recommendations after the intervention [23].

Although students reported a worsening of daytime sleepiness following the intervention, the size of this change should be considered negligible. This is consistent with the finding from a small sample, that changes in sleep behaviour following sleep education did not lead to reduced sleepiness [35]. It might indicate that to successfully address this measure of daytime functioning would require a greater improvement in sleep than was reported. The effect of a school term could partially explain sleepiness levels. The first measurement occurred after the holidays when students would have been more likely to be able to sleep according to preference, and the second towards the end of term when they may have accumulated sleep debt, and consequently sleepiness may have been more prevalent. The timing of assessment may also be pertinent for the only significant change reported in HRQoL, where a small worsening in the quality of students’ interactions with peers and perceptions of their social support was found. Given that HRQoL has been found to be generally stable across adolescence [52] and that any sleep improvements that emerged were small, it is perhaps unsurprising that the intervention did not demonstrate concomitant improvements in this measure.

A different pattern of pre–post results emerged for students who initially reported poor sleep quality and those students whose sleep quality before the intervention could already be considered unproblematic and not indicative of probable insomnia disorder. This corresponds with a study where larger improvements in self-reported sleep patterns were demonstrated by a subclinical group identified with delayed sleep timing [33]. The findings in the current study suggest that sleep education may be more immediately beneficial for those with poor sleep given the improvements these students reported in sleep quality, sleep hygiene and daytime sleepiness. The marked enhancement in their sleep quality is promising, though it should be acknowledged that other factors may have been instrumental. As proposed by Arora and Taheri [53], adolescents who are not experiencing sleep difficulties at baseline are unlikely to change their sleep following an educational programme. It could be inferred that if an evaluation relies solely on a pre–post intervention assessment, then delivering the intervention to all students, regardless of how they are sleeping, would reduce the likelihood that an intervention would be considered successful. However, it does not follow that those who are already sleeping well may not benefit at a later date when facing certain life challenges. Accordingly, it has been put forward that sleep education could provide students with tools to deal with sleep disruption as the first step in preventative sleep medicine [54].

When more detailed sleep monitoring is considered, firstly, the discrepancies between actigraphic and sleep diary measures in TST, SE, SOL, and WASO support previous research [55,56]. Secondly, small putative effects of the intervention were demonstrated in the sub-sample's objective and subjective sleep; for example, weekday lights on was slightly later recorded both by actigraphy (5 min) and sleep diary (6 min). The change in timing is similar to a previous study that found a pre–post intervention delay in actigraphic wake time of 10 min but averaged over seven days in that instance [34]. That sleep-wake timing was largely unchanged may reflect the bioregulatory and environmental challenges (eg, school start times) to sleep faced by adolescents. Sleep pattern variability was remarkably consistent when measured before and after the lessons, highlighting the challenge faced by a sleep education programme to regularise adolescent weekday/weekend timing. Again, it is worth noting that the sub-sample comprised few who reported poor sleep quality at baseline. Students volunteered to take part and this self-selection process and the study's exclusion criteria may have meant that those with a greater potential to experience immediate benefits were less likely to have been included.

Regarding self-reported sleep hygiene recorded in the sleep diary, no significant changes were demonstrated in caffeine intake or electronic media use. However, students did not regularly drink high caffeine items in the evening, minimising the likelihood of a reduced intake. This contrasts with the finding that students used electronic media in the hour before bed on most nights. That this usage continued, suggests that the information and activities on the impact of electronic media use on sleep were not sufficiently persuasive to address the challenge of 24/7 access to technology and the prevalence of social media. Indeed, this challenge was recently highlighted by a study that found that 71.5% of a large cohort of 11 to 12-year-old students in the U.K., reported using at least one screen-based media device within an hour before sleep [57]. When considering why sleep hygiene was found to improve based on responses to the ASHS-r, and yet not from sleep diary reports, it should be noted that the change based on the ASHS-r was small. Potential explanations for the discrepancy in the outcome of these measures are that the questionnaire is based on 24 items whereas individual items are assessed in the diary, different timescales are involved as the questionnaire uses self-report of the previous two weeks rather than one day, and the measures use different response scales. On the other hand, there were encouraging but small improvements during weekdays in reports upon awakening/before sleep in how rested/refreshed and mentally awake they felt. This may indicate that students’ sleep improved and that some used relaxation techniques introduced and practised in the lessons. However, students were not asked whether these techniques were incorporated in their bedtime routine and so interpretation needs to be cautious.

The completion and retention rates for both the survey and sleep monitoring are worthy of attention. Although the study benefitted from a large sample size in the context of school-based sleep education, there was potential for greater numbers to be included in pre–post intervention analyses. Firstly, it could be proposed that the length of the survey, with five questionnaires, may have been influential. The ASHS-r had the lowest completion rate (37%), which may be representative of survey fatigue as participants answered this questionnaire last. A further issue may have been that the survey was completed using pen and paper. An online survey, as an alternative method of data collection, may have reduced the amount of missing data if the failure to respond to an item had been inadvertent. This method has an advantage in that unanswered items can be brought to a participant's attention, who can then be prompted to provide a response if they choose to do so. Secondly, when considering sub-sample sleep monitoring, the percentage of students who met data inclusion requirements (actigraphy: 58%; sleep diary: 51%) may suggest that sustained actigraphy and sleep diary completion can be challenging with this age group. A 14-day protocol was chosen to increase the likelihood that a participant would provide the required five days of sleep on school nights and two days of sleep at weekends. In addition, this time period was considered to improve the potential for the recorded sleep to be representative of students' typical sleep patterns. However, it is not known whether students would have been more or less likely to provide the data required for inclusion in analyses if asked to take part for a shorter time period: for example, a week instead of two weeks. Furthermore, the percentage who provided sufficient data would have been higher if data collection at two schools had not run into the school holidays.

The primary focus of this study and much previous work is on short-term gains for all students. However, if this is the criterion by which sleep education programmes are judged to be successful, it does not take into account how poor sleepers may experience immediate benefits following sleep education nor does it allow for any long-term benefits of increased sleep knowledge to emerge. Establishing evidence to support this contention would be a useful focus for future research. It might promote widespread dissemination of sleep information through inclusion in the health promotion curriculum of schools. Even though schools are not required to provide Personal, Social, Health, and Economic (PSHE) education, it is encouraging that the PSHE Association (the national body for PSHE education in England) recently launched ‘The Sleep Factor’ lesson plans for teachers. An alternative to sleep education for all students would be to develop and evaluate a pragmatic tool kit to improve sleep health for use as an intervention by pastoral/teaching staff only with students reporting poor sleep. Although this option could be easier to fulfil when considering the many competing demands on school curricula and timetables, it should be borne in mind that the remaining individuals might then not receive information that could empower them to maintain good sleep.

Teensleep was funded as a pilot study that was designed to investigate the efficacy and feasibility of the sleep education programme, to find evidence of promise, and to assess whether Teensleep was ready to be evaluated in a subsequent trial. Although the study reported here demonstrated that the Teensleep programme showed evidence of promise in a number of sleep outcomes, any findings should be considered in light of certain limitations. The requirement to run this study as a pilot resulted in the major caveat which concerns the lack of a control group. A control group for comparison would have helped not only to strengthen confidence in the findings but also, in some instances, to aid interpretation; for example, whether the timing of measurement was influential. In addition, a follow-up was not part of the pre–post study design but this would have enabled an investigation into the longevity of any improvements or indeed whether any positive changes emerged at a later date.

Nevertheless, this study is one of the most comprehensive evaluations of a school-based sleep education programme for adolescents to date and has a number of noteable strengths. Firstly, the large sample size in comparison to other sleep education studies increases confidence in our ability to generalise these findings to other adolescent populations. In fact, to our knowledge the only school-based sleep education study to include a greater number of students was that conducted in Hong Kong [49]. Secondly, the evaluation benefitted from a set of measures that included questionnaire responses as well as more comprehensive sleep monitoring using actigraphy and sleep diaries to enable both objective and subjective sleep to be considered.

## 5 Conclusion

Sleep education intuitively seems like a good idea in the teenage years. Indeed, this study suggests that there are educational benefits for all, but that students with poorer sleep quality may demonstrate greater practical benefit. Promisingly, for adolescents with poor sleep, our findings indicate that sleep education is an intervention that could be introduced in schools to facilitate an immediate improvement in sleep. Controlled studies are required to investigate whether or not sleep education, and which components within it, provides long-term benefits as a step towards preventative sleep medicine.

## Credit author statement

**Gaby Illingworth:** Conceptualization, Methodology, Formal analysis, Investigation, Resources, Writing – Original draft, review and editing, Project administration. **Rachel Sharman:** Conceptualization, Methodology, Formal analysis, Investigation, Resources, Data Curation, Writing – review and editing, Project administration. **Christopher-James Harvey:** Conceptualization, Writing – review and editing, Supervision, Project administration, Funding acquisition. **Russell G. Foster:** Conceptualization, Writing – review and editing, Supervision, Funding acquisition. **Colin A. Espie:** Conceptualization, Writing – review and editing, Supervision, Funding acquisition
