# Supplementary material for: The Teensleep study: the effectiveness of a school-based sleep education programme at improving early adolescent sleep
Source: Sleep Med X. 2019 Dec 7;2:100011. doi: 10.1016/j.sleepx.2019.100011 (PMC7790452; doi:10.1016/j.sleepx.2019.100011)
Supplement: Multimedia component 2 [file mmc2.docx]

**Supplementary Table 1**

Means and standard deviations for quiz and survey variables (sleep knowledge, sleep quality, sleep behaviour, daytime sleepiness, sleep hygiene and HRQoL) at Time 1 and Time 2

| **Variable** | **Measure** | **N** | **T1 M (SD)** | **T2 M (SD)** |
| --- | --- | --- | --- | --- |
| Sleep knowledge*** | Quiz | 779 | 9.85 (2.83) | 12.18 (3.27) |
| Sleep quality*** | SCI | 694 | 23.15 (6.55) | 23.87 (5.96) |
| Weekday TST (hh:mm)** | MCTQ | 656 | 7:25 (1:17) | 7:34 (1:13) |
| Weekend TST (hh:mm) | MCTQ | 617 | 8:58 (1:42) | 9:03 (1:41) |
| Weekday SE (%)** | MCTQ | 655 | 93.39 (7.77) | 94.12 (5.85) |
| Weekend SE (%) | MCTQ | 616 | 95.45 (5.32) | 95.46 (5.68) |
| Weekday SOL (min) | MCTQ | 770 | 29.89 (31.87) | 27.78 (30.76) |
| Weekend SOL (min) | MCTQ | 746 | 25.84 (32.14) | 25.83 (32.45) |
| Weekday lights out (hh:min) | MCTQ | 721 | 22:56 (1:07) | 22:52 (1:07) |
| Weekend lights out (hh:min)** | MCTQ | 685 | 00:22 (1:42) | 00:14 (1:38) |
| Weekday lights on (hh:min)** | MCTQ | 798 | 6:49 (0:33) | 6:52 (0:37) |
| Weekend lights on (hh:min) | MCTQ | 760 | 9:42 (1:44) | 9:40 (1:43) |
| Daytime sleepiness* | CASQ | 727 | 35.73 (9.71) | 36.27 (9.36) |
| Sleep hygiene: |  |  |  |  |
| Sleep hygiene total score** | ASHS-r | 562 | 4.19 (.68) | 4.26 (.65) |
| Physiological | ASHS-r | 562 | 4.38 (.87) | 4.40 (.81) |
| Behavioural arousal | ASHS-r | 562 | 3.21 (1.21) | 3.27 (1.17) |
| Cognitive/emotional* | ASHS-r | 562 | 4.15 (1.07) | 4.24 (1.02) |
| Sleep environment | ASHS-r | 562 | 5.10 (.86) | 5.15 (.82) |
| Sleep stability** | ASHS-r | 562 | 3.12 (1.23) | 3.27 (1.26) |
| Daytime sleep | ASHS-r | 562 | 5.18 (1.12) | 5.21 (1.09) |
| HRQoL: |  |  |  |  |
| Physical wellbeing | KS-27 | 710 | 44.03 (8.63) | 43.61 (8.33) |
| Psychological wellbeing | KS-27 | 710 | 44.44 (9.38) | 44.25 (9.39) |
| Autonomy & parent relations | KS-27 | 710 | 47.70 (10.60) | 47.54 (10.52) |
| Social support & peers*** | KS-27 | 710 | 48.76 (9.96) | 46.63 (10.52) |
| School environment | KS-27 | 710 | 44.66 (8.52) | 44.59 (9.08) |

*Note:* Discrepancies in sample size are due to missing responses; SCI = Sleep Condition Indicator; MCTQ = Munich Chronotype Questionnaire; CASQ = Cleveland Adolescent Sleepiness Questionnaire; ASHS-r = Adolescent Sleep Hygiene Scale – revised; HRQoL = Health-related quality of life; KS-27 = KIDSCREEN-27.
**p* < .05, ***p* <.01, ****p* <.001

**Supplementary Table 2**

Means and standard deviations for sub-sample actigraphy and sleep diary variables at Time 1 and Time 2

| **Variable** | **Actigraphy (*n* = 84)** | | **Sleep diary (*n* = 74)** | |
| --- | --- | --- | --- | --- |
|  | **T1 M (SD)** | **T2 M (SD)** | **T1 M (SD)** | **T2 M (SD)** |
| Weekday TST (hh:mm) | 7:03 (0:39) | 7:02 (0:39) | 7:46 (1:05) | 7:51 (1:02) |
| Weekend TST (hh:mm) | 7:59 (0:55) | 8:00 (1:04) | 8:46 (1:10) | 8:44 (1:07) |
| Weekday SE (%) | 84.95 (4.20) | 84.84 (4.17) | 93.04 (7.01)^a^ | 94.19 (5.03)^a^ |
| Weekend SE (%) | 84.59 (4.82) | 84.53 (5.12) | 93.29 (7.64)^a^ | 93.33 (7.23)^a^ |
| Weekday SOL (min) | 13.71 (10.47)^b^ | 14.25 (10.19)^b^ | 27.08 (23.04) | 23.59 (17.83) |
| Weekend SOL (min) | 13.03 (13.68)^b^ | 15.55 (21.92)^b^ | 21.59 (18.58) | 23.69 (18.23) |
| Weekday WASO (min) | 59.92 (19.71) | 60.55 (20.56) | 14.28 (25.36) | 8.13 (14.82)** |
| Weekend WASO (min) | 70.86 (23.27) | 70.99 (23.67) | 12.37 (22.23) | 8.61 (13.59) |
| Weekday lights out (hh:min) | 22:31 (00:50) | 22:38 (00:56) | 22:28 (00:53) | 22:36 (01:04) |
| Weekend lights out (hh:min) | 23:22 (01:06) | 23:26 (01:16) | 23:11 (01:14) | 23:12 (01:22) |
| Weekday lights on (hh:min) | 06:51 (00:33) | 06:56 (00:37)** | 06:43 (00:35) | 06:49 (00:37)** |
| Weekend lights on (hh:min) | 08:49 (01:18) | 08:54 (01:35) | 08:32 (01:17) | 08:29 (01:35) |
| Weekday lights out variability (min) | 23 (13) | 27 (15)* | 23 (15) | 27 (19) |
| Weekend lights out variability (min) | 66 (43) | 66 (44) | 67 (43) | 61 (57) |
| Weekday lights on variability (min) | 13 (10) | 12 (13) | 12 (13) | 15 (17) |
| Weekend lights on variability (min) | 121 (63) | 123 (73) | 116 (64) | 114 (71) |

*Note:* ^a^ Sleep diary SE capped at 100%; ^b^ Actigraphic SOL: Weekday *n* = 82; Weekend *n* = 79.
**p* < .05, ***p* <.01

**Supplementary Table 3**

Means and standard deviations for sub-sample sleep diary variables of sleep hygiene, sleep need, sleep quality and cognitive/physical factors at Time 1 and Time 2 (*n* = 74)

| **Variable** | **Weekday T1**  **M (SD)** | **Weekday T2 M (SD)** | **Weekend T1 M (SD)** | **Weekend T2 M (SD)** |
| --- | --- | --- | --- | --- |
| Number of high caffeine item days (%)^a^ | 5.81 (15.82) | 5.35 (14.89) | 4.73 (13.80) | 7.32 (21.05) |
| Number of low caffeine items days (%)^a^ | 42.97 (32.67) | 46.78 (34.60) | 47.97 (37.44) | 52.93 (37.56) |
| Number of electronic media use days (%)^a^ | 93.38 (12.03) | 90.75 (20.26) | 96.85 (9.53) | 94.14 (14.25) |
| Number of nights wanted to sleep longer (%)^a^ | 70.87 (33.58) | 68.50 (37.23) | 38.96 (37.35) | 37.50 (38.87) |
| Number of minutes wanted to sleep longer per day | 77.94 (67.62) | 73.35 (61.94) | 37.62 (49.42) | 39.19 (50.06) |
| How rested/refreshed do you feel this morning? | 1.81 (.74) | 1.97 (.69)* | 2.34 (.70) | 2.41 (.74) |
| How awake do you feel this morning? | 1.74 (.69) | 1.82 (.64) | 2.30 (.72) | 2.39 (.73) |
| How mentally awake were you in bed last night? | 1.59 (.79) | 1.41 (.85)* | 1.47 (.84) | 1.34 (.83) |
| How physically tense were you in bed last night? | 1.01 (.70) | .93 (.73) | .91 (.75) | .90 (.72) |

*Note:* ^a^ The number of days where students consumed caffeine, used electronic media or wanted to sleep longer are reported as a percentage of days provided.
**p* < .05
